# Detection of *SQSTM1/P392L* post-zygotic mutations in Paget’s disease of bone

**DOI:** 10.1007/s00439-014-1488-3

**Published:** 2014-09-21

**Authors:** Sabrina Guay-Bélanger, Sylvain Picard, Edith Gagnon, Jean Morissette, Ethel S. Siris, Philippe Orcel, Jacques P. Brown, Laëtitia Michou

**Affiliations:** 1 CHU de Québec Research Centre, Rhumatologie-R4774, CHU de Québec, 2705 boulevard Laurier, Québec, QC G1V 4G2 Canada; 2 Division of Rheumatology, Department of Medicine, Université Laval, Québec, QC Canada; 3 Department of Pathology, CHU de Québec, Québec, QC Canada; 4 Columbia University Medical Centre, New York, NY USA; 5 Pôle appareil locomoteur, service de rhumatologie B, hôpital Lariboisière AP-HP, Paris, France; 6 Department of Rheumatology, CHU de Québec, Québec, QC Canada

## Abstract

Paget’s disease of bone (PDB) is transmitted, in one-third of cases, in an autosomal dominant mode of inheritance with incomplete penetrance. The *SQSTM1/P392L* germinal mutation is the most common mutation associated with PDB. Given the focal nature of PDB, one team of investigators showed that *SQSTM1/P392L* somatic mutations could occur in pagetic bone lesions in the absence of germinal mutations detectable in the peripheral blood. The objectives of this study were to develop a reliable method to detect *SQSTM1/P392L* post-zygotic mutations, by optimizing a polymerase chain reaction (PCR)-clamping method reported to be effective in detecting post-zygotic mutations in peripheral blood from patients with fibrous dysplasia; and to evaluate the frequency of this post-zygotic mutation in PDB patients. We used a locked nucleic acid (LNA) specifically designed for the *SQSTM1/P392L* mutation, which blocks the wild-type allele amplification during the PCR. DNA from 376 pagetic patients and 297 controls, all without any *SQSTM1/P392L* germinal mutation, was analyzed. We found that 4.8 % of PDB patients and 1.4 % of controls were carriers of this post-zygotic mutation [*p* = 0.013, OR 3.68 (1.23; 11.00)]. PDB patient carriers of a post-zygotic mutation had a lower number of affected bones and Renier’s index than patients carrying a germinal mutation, suggesting a lower disease extension. We also demonstrated that this post-zygotic mutation was restricted to the monocytic lineage. These results confirmed that LNA PCR clamping is effective for the detection of *SQSTM1/P392L* post-zygotic mutations, which may occur in patients with PDB.

**Electronic supplementary material:**

The online version of this article (doi:10.1007/s00439-014-1488-3) contains supplementary material, which is available to authorized users.

## Introduction

Paget’s disease of bone (PDB) is the second most frequent metabolic bone disorder after osteoporosis, affecting up to 3 % of adults over 55 years of age (Collet et al. 2007). PDB is characterized by focal increases in bone turnover, affecting one or several bones, and resulting in abnormal bone architecture and weakened bone strength (Michou et al. 2006). Although the exact etiology of the disease is still unknown, the genetic component plays an important role in its pathophysiology. In fact, one-third of patients have a familial form of this disease, which is transmitted in an autosomal dominant mode of inheritance with incomplete penetrance (Michou and Brown 2011). Several chromosomal regions have been linked to PDB, confirming its genetic heterogeneity. The *5q35*-*qter* locus was first reported in the French-Canadian population, and led to the identification of the first germinal mutation associated with PDB. This substitution of a cytosine to a thymine at position 1,215 in the *Sequestosome 1* (*SQSTM1*) gene results in the substitution of the proline at position 392 to a leucine (*p.Pro392Leu,* called here *P392L*) in the p62 protein (Laurin et al. 2001, 2002). As of now, more than 20 mutations in the *SQSTM1* gene have been described (Chung and Van Hul 2011; Hocking et al. 2002; Morissette et al. 2006). *SQSTM1* mutations are reported in similar frequencies across countries: *SQSTM1* mutations are present in 24.5 % of patients with a familial history of PDB and 10.5 % of unrelated PDB patients in Australia (Rea et al. 2009). In Italy, 36.9 and 9.7 % of patients with or without a familial history of PDB, respectively, were carriers of a *SQSTM1* mutation (Gennari et al. 2010), and similar frequencies were also observed in United Kingdom and New Zealand (Cundy et al. 2011; Hocking et al. 2004). In United States, *SQSTM1* mutations are present in 20.5 % of patients with a familial history of PDB, while none were reported in sporadic patients (Rhodes et al. 2008). However, the *SQSTM1/P392L* mutation remains the most frequent mutation linked to PDB, with an overall frequency of 23.6 % in familial cases, and 7.1 % in unrelated patients (Morissette et al. 2006). In the French-Canadian population, only the *SQSTM1/P392L* mutation is present, with frequencies of 46 % in familial cases, and 16 % in unrelated patients (Morissette et al. 2006). Environmental factors also play an important role in PDB pathogenesis, in particular viral infections, as pagetic osteoclasts frequently express the measles virus nucleocapsid protein (MVNP) (Teramachi et al.^ 2014^).

PDB has an asymmetrical distribution and remains highly localized to affected bones, patients rarely developing new lesions after diagnosis (Roodman and Windle 2005). To explain this focal nature, some authors suggested, and then showed, that somatic mutations in the *SQSTM1* gene could occur in pagetic bone lesions. Indeed, one team has reported a *SQSTM1/P392L* mutation in affected bone of two unrelated patients with PDB, but not in their peripheral blood, suggesting a somatic origin for these mutations (Merchant et al. 2009). Another independent study failed to detect any somatic mutations of the *SQSTM1* gene in osteoblasts and bone marrow cells culture of PDB-affected patients (Matthews et al. 2009). Thus, these results may suggest that *SQSTM1/P392L* somatic mutations can be detected in pagetic bone lesions, but not in bone marrow of PDB patients.

Fibrous dysplasia (FD) is a focal bone disorder with an asymmetrical mono- or polyostotic distribution, like PDB. FD is caused by post-zygotic mutations in the *Guanine Nucleotide*-*binding Protein Alpha*-*Stimulating activity* (*GNAS*) gene, encoding for the α-subunit of the Gs protein. Post-zygotic mutations are somatic mutations occurring during the early development of the zygote, resulting in a mosaic distribution of normal and mutant cells in tissues or organs of affected individuals (Weinstein 2006). Most of the time, the mutation reported in FD is a substitution of the arginine at position 201 of the Gs protein to a histidine or a cysteine, and, rarely, to a glycine or a leucine (Michou and Brown 2010). Since the detection of these post-zygotic mutations is difficult without practicing a bone biopsy, a sensible polymerase chain reaction (PCR)-clamping method has been developed. This technique used a peptide nucleic acid (PNA), a primer that specifically blocks the amplification of the wild-type allele; thereby, improving the sensitivity to detect the mutant allele, even if this one was present in low copy number in DNA from peripheral blood of affected individuals (Lietman et al. 2005).

Given the similarities in the skeletal distribution between FD and PDB and the possible presence of *SQSTM1/P392L* somatic mutations in pagetic bone lesions, we hypothesized that the optimization of the PCR method, developed to detect *GNAS* post-zygotic mutations in FD, would allow us to detect *SQSTM1/P392L* post-zygotic mutations in the peripheral blood of patients with PDB. Thus, the objectives of this study were to develop a reliable method to detect *SQSTM1/P392L* post-zygotic mutations and to evaluate the frequency of this post-zygotic mutation in PDB.

## Materials and methods

### Individuals

This study was approved by the CHU de Québec Ethics Committee, and all participants, affected or not, signed a consent form before entering the study. A complete bone evaluation, including total serum alkaline phosphatase (sALP) measurement, skull and pelvis X-rays and a whole-body bone scan was performed for each patient. The criteria used to diagnose PDB were: (1) an increase in total sALP level and/or (2) a typical aspect of PDB on the bone X-rays and/or (3) an abnormal whole-body bone scan, as previously reported (Laurin et al. 2001). Patients with FD were diagnosed on a typical bone aspect by the use of imaging. All patients with FD studied here were French-Canadians. The mean age was 43.5 ± 14.6 years, 50.0 % of them had only one bone affected, and 60.0 % were male. Patients with PDB originated from three different countries: Canada (French-Canadians from the province of Quebec), France and United States (New York city area). Either they suffered from a familial form of PDB or they were considered as unrelated affected individuals. All participants studied were screened for germinal mutations in exons 7 and 8 of the *SQSTM1* gene, and none of them were carrier of a *SQSTM1* gene mutation. In the French-Canadian patients, the mean age at the time of the study was 80.3 ± 10.9 years, 57.9 % were male and 45.2 % had a monostotic disease (Laurin et al. 2002; Morissette et al. 2006). In the French population, the mean age of PDB patients was 70.7 ± 14.0 years, 56.3 % were male and 31.3 % had a monostotic disease. Finally, in the New York city area population, 44.4 % were male and 60.3 % had a monostotic bone involvement (the mean age was not available for this population) (Michou et al. 2010, 2011). Clinical characteristics of pagetic patients, including total sALP (expressed as the number of times the midpoint of normal range to normalize results between patients), the age at PDB diagnosis, the number of bone sites affected by PDB and the skeletal extension calculated by the Rénier’s index, were collected (Renier et al. 1995). Controls were healthy individuals from the French-Canadian population without any personal or familial history of PDB based on a questionnaire, and with normal total sALP levels at inclusion. For three of them, a bone scan was performed (see results section). The mean age of these healthy individuals at the time of the study was 76.7 ± 10.9 years, and 28.4 % were male. For each participant, DNA from peripheral blood mononuclear cells (PBMCs) was extracted according to standard procedures.

### Development of the PCR-clamping method for the *GNAS*/*R201L* mutation in FD patients

Despite good results have been obtained in detecting somatic mutations in FD using a PNA in the literature (Lietman et al. 2005), the LNA has many advantages: the LNA has a higher stability when bonded to DNA, a higher affinity for complementary DNA sequences than PNA or than DNA itself, and a better sensitivity to the presence of mismatched bases (Braasch and Corey 2001). For all these reasons, we preferred, in our study, optimizing the PCR-clamping method by the use of an LNA instead of a PNA. We optimized this PCR-clamping technique in 10 patients with FD, in accordance with the literature (Lietman et al. 2005). The LNA was specifically designed for the *GNAS/R201L* mutation and synthesized by Exiqon (Woburn, MA, USA) (5′-TTCGCTGCCGTGTCCTGAC-3′). A portion of exon 8 of the *GNAS* gene was amplified by PCR using the following primers: forward 5′-CACCCCACGTGTCTTTCTTT-3′ and reverse 5′-CACAGCATCCTACCGTTGAA-3′. Each 20 µL PCR contained 40 ng of DNA, 1× PCR buffer, 1.5 mM MgCl_2_, 0.4 µM of each primer, 0.2 mM of dNTPs, 1 M of betaine, 0.4 U of JumpStart Taq DNA Polymerase and 5 µM of the specific LNA. PCR conditions were an initial denaturation at 95 °C for 5 min, followed by 38 cycles at 95 °C for 30 s (denaturation step), 74 °C for 30 s (LNA hybridization step), 55 °C for 30 s (primer annealing step) and 72 °C for 30 s (extension step), and an additional extension step was performed at 72 °C for 8 min. Amplification products were purified and sequenced with Big Dye Deoxy Terminator v 3.1 Cycle (Applied Biosystems) on an ABI 3730xl sequencer at the Plateforme de séquençage et de génotypage des génomes du Centre de recherche du CHU de Québec. The DNA sequences obtained were analyzed with Staden package version 1.6 (Staden 1996). All chromatograms were independently analyzed by two readers (SGB, LM). In case of discordant interpretation, the PCR clamping followed by the sequencing analysis was repeated. All sequences showing a post-zygotic mutation were done at least twice to confirm the results.

### Optimization of the PCR-clamping method for the *SQSTM1/P392L* mutation in PDB patients

In this study, we only investigated the *SQSTM1/P392L* mutation, the most common mutation associated with PDB so far, since this mutation occurs at a hypermutable CpG dinucleotide sequence, a mutational hotspot (Laurin et al. 2002; Morissette et al. 2006). We used an LNA specifically designed for this *SQSTM1/P392L* mutation, which was synthesized by Exiqon (Woburn, MA, USA) (5′-CAGCCGCGGGTCAGC-3′). To confirm the LNA’s ability to block the wild-type allele amplification, we analyzed DNA from three types of control individuals: one healthy individual non-carrier of a *SQSTM1/P392L* germinal mutation, one pagetic patient heterozygous for the *SQSTM1/P392L* germinal mutation, and one patient homozygous for this germinal mutation. A portion of exon 8 of the *SQSTM1* gene was amplified using the following primers: forward 5′-TGGCTAACTGGCCTGTTCTT-3′ and reverse 5′-AATGGCTTCTTGCACCCTAA-3′. PCR mix and conditions were the same as those used for the detection of the *GNAS/R201L* post-zygotic mutation. Given the small amount of amplified fragments in the presence of LNA, we performed a nested PCR using the following primers: forward 5′-TACAGGGAAAGCAGGTCCAC-3′ and reverse 5′-TCCTGGAAGAAGGCAGAGAA-3′, and standard PCR conditions. Amplification products were purified, sequenced, and analyzed as previously mentioned. Once the LNA PCR-clamping technique was accurate, we analyzed DNA from 297 French-Canadian PDB patients, of whom 75 had a familial form of the disease, 16 PDB patients from the French population, of whom five suffered from a familial form of the disease, 63 unrelated PDB patients from the New York city area population, and 297 French-Canadian healthy controls, all non-carrier of a *SQSTM1/P392L* germinal mutation. Finally, to ensure that the post-zygotic mutations detected were not false positives due to the PCR method’s limits, we analyzed DNA from 19 individuals diagnosed with a rare bone disease and who are not likely to carry a *SQSTM1/P392L* post-zygotic mutation.

### Distribution of the *SQSTM1/P392L* post-zygotic mutation in blood and saliva tissues of PDB patients

Blood samples (50 mL) were collected from three PDB patient carriers of a *SQSTM1/P392L* post-zygotic mutation, one PDB patient heterozygous for the *SQSTM1/P392L* germinal mutation and one PDB patient non-carrier of any mutation within the *SQSTM1* gene. PBMCs were isolated by Ficoll density gradient and dextran sedimentation. The isolated PBMCs were then centrifuged and incubated 20 min in PBS/0.2 % BSA with the following antibodies: PercpCy5.5-conjugated anti-human CD45, PE-conjugated anti-human CD14, FITC-conjugated anti-human CD3, APC-conjugated anti-human CD19 and V450-conjugated anti-human CD56, according to the manufacturer’s protocol. Monocytes and lymphocytes were further sorted using the BD SORP FACSAria II cytometer at the Plateforme d’imagerie et cytométrie du Centre de recherche du CHU de Québec. Cells considered as monocytes were CD45^+^CD14^+^ cells, and those considered as lymphocytes were CD45^+^CD3^+^ (T lymphocytes), CD45^+^CD19^+^ (B lymphocytes) and CD45^+^CD56^+^ (natural killers) cells. Saliva samples (2 mL) were collected from two PDB patient carriers of a *SQSTM1/P392L* post-zygotic mutation using the Oragene-DNA kit, provided by DNA Genotek (Ottawa, ON, Canada). DNA was extracted from monocytes, lymphocytes, and saliva separately using standard procedures, and the LNA PCR-clamping method was performed as previously described to detect *SQSTM1/P392L* post-zygotic mutations in these cell populations.

### Distribution of the *SQSTM1/P392L* post-zygotic mutation in bone tissue of a PDB patient

A transiliac bone biopsy was performed on the only patient carrier of a *SQSTM1/P392L* post-zygotic mutation who had a pelvic site involved with PDB, using a Rochester trephine (inner diameter 7–8 mm). Bone biopsy was bi-sectioned with low ISOMET speed saw (Buehler, Canada) and one section was frozen at −80 °C for laser microdissection of osteoclasts, before DNA extraction and sequencing. The other part of the biopsy was fixed in Phosphate buffered 10 % formaldehyde prior to being decalcified in 14 % EDTA for 3 days. Then, this bone sample was embedded in paraffin. A 5-μm-thick section was sectioned with a Leica RM2245 microtome and H&E stained to confirm that the bone biopsy was indeed in a pagetic area.

### Estimation of the copy number of the *SQSTM1/P392L* post-zygotic mutation

A fluorescent-based Realtime PCR quantification was performed on 10 ng of genomic DNA from 18 PDB patients carrier of a *SQSTM1/P392L* post-zygotic mutation, two PDB patient carriers of a homozygous *SQSTM1/P392L* germinal mutation, two PDB patients heterozygous for this same mutation, and 15 individuals non-carrier of any mutation. We used the LightCycler 480 (Roche Diagnostics, Mannheim, DE), and reagent LightCycler 480 SYBRGreen I Master (Roche Diagnostics, Indianapolis, IN, USA) as described by the manufacturer, with 2 % DMSO. A portion of exon eight of the *SQSTM1* gene was amplified using two specific forward LNA-enhanced qPCR primers synthetized by Exiqon (Woburn, MA, USA): 5′-TGTTTCGGCAGAGGCTGACC + C-3′ for the wild-type allele, and 5′-TGTTTCGGCAGAGGCTGACC + T-3′ for the mutant allele. The reverse primer was the same for both reactions and was synthesized without LNA (5′-TCCGATGTCATAGTTCTTGGTCTGC-3′). PCR conditions were 40 cycles at 90 °C for 10 s (denaturation), 65 °C for 10 s (annealing), 72 °C for 14 s (elongation) and 80 °C for 5 s (reading). A melting curve was performed to assess non-specific signal, and a standard curve was established using known amount of purified PCR products. Calculation of the number of copies of each mRNA was performed using second derivative method and a standard curve of Cp versus logarithm of the quantity, using the LightCycler 480 v1.5 program provided by the manufacturer (Roche Diagnostics, Mannheim, DE). Finally, the percentage of the mutant allele T versus wild-type allele C was calculated for each sample. All manipulations and analyses were performed at the Plateforme d’expression génique du Centre de recherche du CHU de Québec.

### Statistical analyses

Frequencies of the *SQSTM1/P392L* post-zygotic mutation between cases and controls were compared using a Chi-square test, with odds ratio (OR) and 95 % confidence interval (95 %CI) calculations. For the phenotype–genotype associations, we compared PDB patients with post-zygotic mutations to patients with germinal mutations and without any mutation within the *SQSTM1* gene, for the following items: total sALP levels, age at diagnosis, number of affected bones, and Renier’s index. Analyses relied on Student *t* test for continuous variables, and Chi-square or Fisher exact tests when appropriate for nominal values. All analyses were performed using IBM SPSS Statistics 21 and a *p* value of <0.05 was considered statistically significant.

## Results

### Development of the PCR-clamping method for the *GNAS*/*R201L* mutation in FD patients

**Fig. 1:**
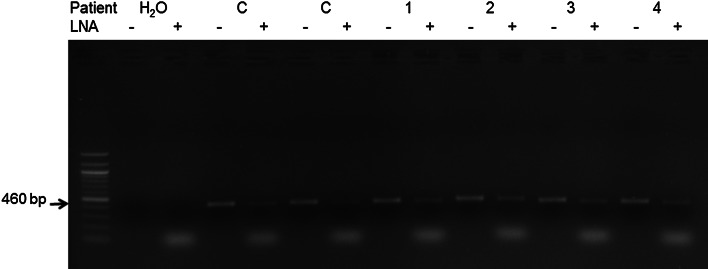
LNA PCR-clamping method for the *GNAS/R201L* mutation in patients with fibrous dysplasia (FD). PCR was performed in the absence (−) or in the presence (+) of the LNA. DNA from FD patients (lanes labeled* 1–4*) and healthy controls (lanes labeled *C*) was used. A band of 460 bp was present when PCR was performed in absence of the LNA, and the intensity was reduced by the addition of the LNA in the PCR mix, as expected. PCR fragments were loaded on a 1 % agarose gel. The *water lane* represented a control without DNA, and the absence of a PCR band indicated that there was no contamination in this experience

**Fig. 2:**
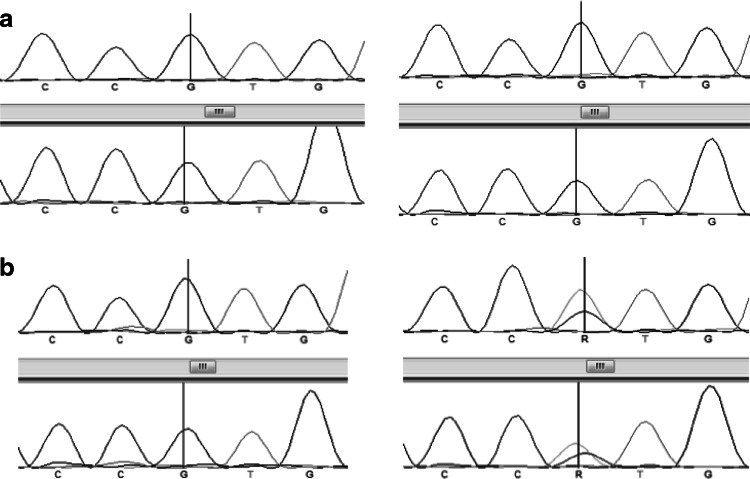
Sequencing analysis with and without the use of the LNA PCR-clamping method in patients with fibrous dysplasia (FD). *Forward* and *reverse* short nucleotide sequence analysis of the region covering the *GNAS/R201L* mutation is presented. *Left panel* shows the sequences in absence of the LNA, and *right panel* shows the DNA sequence in presence of the LNA. **a** DNA sequence of a FD patient non-carrier of a *GNAS/R201L* post-zygotic mutation. Regardless of the presence of the LNA, only the wild-type *G* allele was amplified. **b** DNA sequence of the patient FD-02, who is carrier of a *GNAS/R201L* post-zygotic mutation. In absence of the LNA, only the wild-type G allele was amplified, while in presence of the LNA, both wild-type G and mutant A allele were amplified

The LNA PCR-clamping method was developed with DNA from PBMCs of ten patients with FD and two healthy controls. Figure 1 shows that the LNA specifically designed for the *GNAS/R201L* mutation was effective to block the wild-type allele amplification. Direct sequencing of the PCR products in presence of the LNA showed that one individual was carrier of a *GNAS/R201L* post-zygotic mutation, which was not detected in the absence of the LNA (Fig. 2). The FD patient carrier of the *GNAS/R201L* post-zygotic mutation, who was the only one to have a McCune-Albright syndrome, also seemed to have a more extensive disease than non-mutated FD patients (see FD-02, Table 1). In fact, he had 11 FD-affected bones, while the mean number in this cohort was 3.2 ± 3.4 affected bones. These results suggested that the *GNAS/R201L* post-zygotic mutation can be detected with this PCR-clamping technique in FD.

**Table 1:** Description of the main clinical characteristics of patients (*n* = 10) with fibrous dysplasia (FD)

Patient ID	Sex	Age at diagnosis (years)	Affected bones	Extraosseous involvements or complications of FD
FD-01	M	40	Sphenoid	None
FD-02^a^	M	15	Skull, right maxillary, mandible, rib cage, both humeri, right radius, left ulna, right pelvis, right femur, right tibia	McCune Albright syndrome, back and neck *café au lait* skin lesions, hypophosphatemia, erectile dysfunction
FD-03	M	26	Right temporal region	Stenosis of the right external auditory canal
FD-04	M	24	Left mastoid and occipital regions, sphenoid, left mandible	Bacterial meningitis, complete deafness on the left side, infertility
FD-05	M	45	Both tibias	Mazabraud’s syndrome (left calf and right thigh myxoma in addition to FD)
FD-06	F	28	Left mastoid and occipital regions	None
FD-07	F	43	Skull, D9 and L2 vertebrae, left ischion, left sacroiliac region, both femurs	None
FD-08	M	24	Sphenoid	None
FD-09	F	57	Right frontal region, ethmoid, nasal bone, malar region, right mandible	None
FD-10	F	26	Skull	None

^a^FD-02 was found to be carrier of a *GNAS/R201L* post-zygotic mutation in our study

### Optimization of the PCR-clamping method for the *SQSTM1/P392L* mutation in PDB patients

**Fig. 3:**
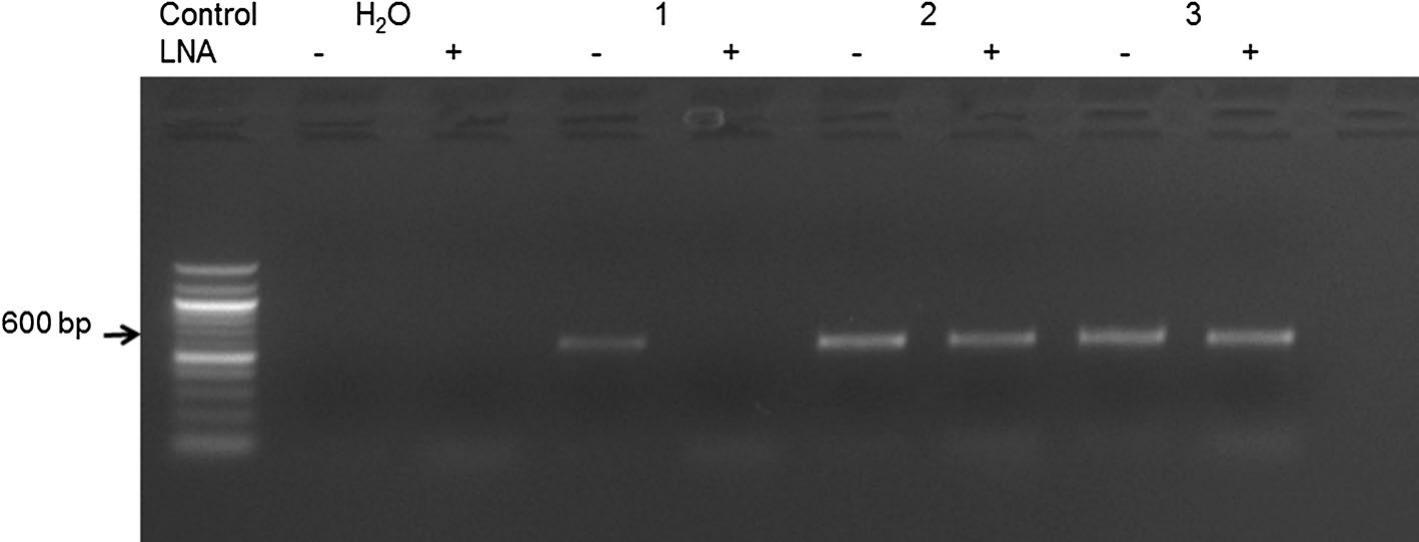
Optimization of the LNA PCR-clamping method for the *SQSTM1*/*P392L* mutation in patients with Paget’s disease of bone (PDB). PCR was performed in absence (−) or in the presence (+) of the LNA. DNA from PBMCs of a non-mutated healthy individual (lane labeled* 1*), a patient with a heterozygous genotype for the *SQSTM1*/*P392L* germinal mutation (lane labeled* 2*), and a patient with a homozygous genotype for the *SQSTM1*/*P392L* germinal mutation (lane labeled* 3*) was used. A band of 600 bp was present when PCR was performed in absence of the LNA. For the mutated individuals, this band was still present when the LNA was added in the PCR mix, while completely absent for the non-mutated healthy individual. PCR fragments were loaded on a 1 % agarose gel. The water lane represented a control without DNA, and the absence of a PCR band indicated that there was no contamination in this experience

**Fig. 4:**
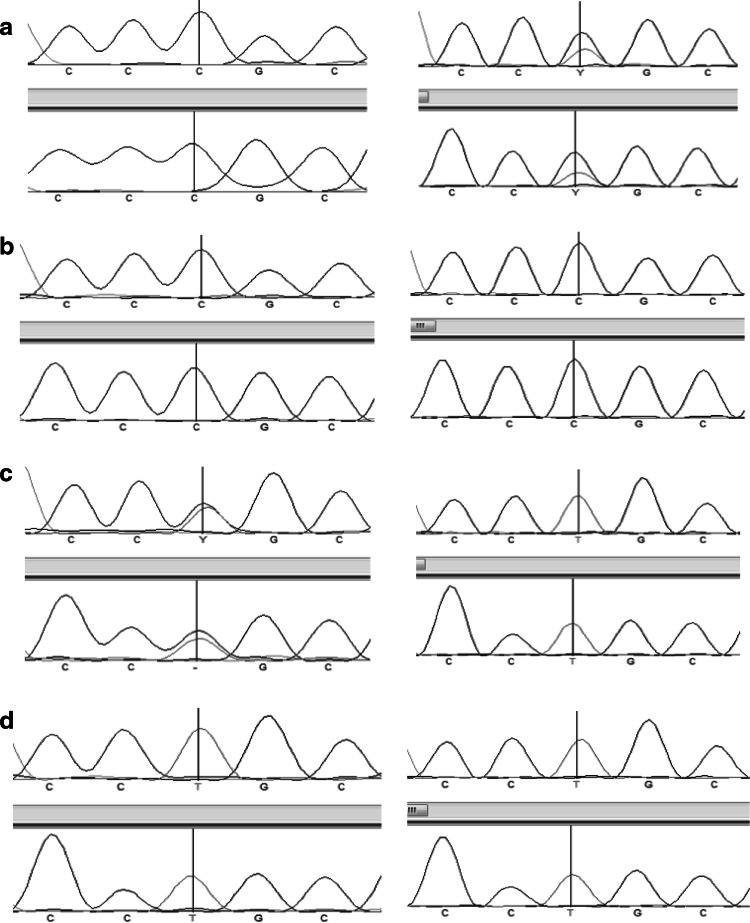
Sequencing analysis with and without the utilization of the LNA PCR-clamping method in patients with Paget’s disease of bone (PDB). Forward and reverse short nucleotide sequence analysis of the region covering the *SQSTM1/P392L* mutation is presented. *Left panel* shows the sequences in absence of the LNA, and *right panel* shows the DNA sequence in presence of the LNA. **a** DNA sequence of a PDB patient carrier of a *SQSTM1/P392L* post-zygotic mutation. In absence of the LNA, only the wild-type* C* allele was amplified, while in presence of the LNA, both wild-type C and mutant T allele were amplified. **b** DNA sequence of a non-mutated healthy individual. Regardless of the presence of the LNA, only the wild-type C allele was amplified. **c** DNA sequence of a PDB patient carrier of a heterozygous *SQSTM1/P392L* germinal mutation. In absence of the LNA, both wild-type C and mutant T allele were amplified; while in presence of the LNA, only the mutant T allele was amplified. **d** DNA sequence of a PDB patient carrier of a homozygous *SQSTM1/P392L* germinal mutation. Regardless of the presence of the LNA, only the mutant T allele was amplified, as expected

The PCR performed on DNA from PBMCs of three control individuals confirmed that the LNA specifically designed for the *SQSTM1/P392L* mutation was very effective to block the wild-type allele amplification (Fig. 3). Then, the PCR-clamping method was performed on DNA from PBMCs of a subset of patient non-carriers of a germinal mutation to search for *SQSTM1/P392L* post-zygotic mutations. Direct sequencing of PCR products showed that one patient in this subset was carrier of a *SQSTM1/P392L* post-zygotic mutation (Fig. 4). These results suggested that this LNA PCR-clamping method was effective to detect *SQSTM1/P392L* post-zygotic mutations in peripheral blood of pagetic patients.

### Prevalence of the *SQSTM1/P392L* post-zygotic mutation in PDB patients

After verifying the accuracy and precision of the PCR-clamping method, we evaluated the prevalence of the *SQSTM1/P392L* post-zygotic mutation in 376 PDB-affected patients from three distinct populations (Quebec, France and New York city area) and 297 French-Canadian healthy individuals, all non-carriers of a *SQSTM1/P392L* germinal mutation. In the French-Canadian population, we detected a *SQSTM1/P392L* post-zygotic mutation in 17 (5.7 %) patients with PDB (Table 2). Among them, five unrelated patients had a familial autosomal dominant form of the disease, but with absence of germinal or post-zygotic *SQSTM1/P392L* mutation in their relatives. In the French population, one (6.3 %) patient with an autosomal dominant familial form of PDB was also carrier of a *SQSTM1/P392L* post-zygotic mutation (Table 2). We did not detect this post-zygotic mutation in patients with PDB from the New York city area population. Also, this post-zygotic mutation was not present in non-pagetic patients with a rare bone disease, further supporting that the post-zygotic mutations observed in PDB patients were not false positives due to the limits of the PCR method. Overall, in the three cohorts, we found 18 (4.8 %) PDB patients carriers of a *SQSTM1/P392L* post-zygotic mutation, the difference with healthy controls being statistically significant [*p* = 0.013, OR 3.68 (1.23; 11.00)]. Surprisingly, we found four (1.4 %) carriers of a *SQSTM1/P392L* post-zygotic mutation in a priori healthy French-Canadian individuals, based on questionnaire and total sALP measurements at inclusion. We were able to perform clinical examinations and whole-body bone scan in three of these healthy individuals to search if they had asymptomatic PDB. The clinical exam of the first one, an 81-year-old woman, demonstrated a frank hypertrophy of the internal extremity of the right clavicle, consistent with a diagnosis of PDB, but the bone scan only demonstrated degenerative lumbar and knee fixations, and no X-rays were available. The second healthy control was a 70-year-old man, without any clinical evidence of PDB. His bone scan did not show any fixation suggestive of PDB. The third control carrier of a post-zygotic mutation was a 54-year-old woman who reported permanent pain of the right parietal bone, but her bone scan and skull X-rays did not show any evidence of PDB.

**Table 2:** Description of the main clinical characteristics of pagetic patients (*n* = 18) carriers of a *SQSTM1/P392L* post-zygotic mutation

Patient ID	Population	Family history	Sex	Age at diagnosis (years)	Total sALP levels^a^	Number of affected bones	Affected bones	Renier’s index (%)
PDB-01	French-Canadian	No	M	53	1.95	3	Right femur, right clavicle, right frontal region	4.82
PDB-02	French-Canadian	No	F	68	11.99	2	Skull, sacrum	13.00
PDB-03	French-Canadian	No	M	65	7.73	3	Left pelvis, left femur, lumbar vertebra	8.35
PDB-04	French-Canadian	No	F	30	1.48	1	Left tibia	5.00
PDB-05	French-Canadian	No	M	42	11.07	1	Sacrum	2.00
PDB-06	French-Canadian	No	M	49	2.32	2	Right femur, right scapula	7.80
PDB-07	French-Canadian	No	F	57	2.19	1	Right femur	9.00
PDB-08	French-Canadian	No	F	63	2.59	3	Skull, right pelvis, right femur	20.00
PDB-09	French-Canadian	No	M	64	1.28	2	Sacrum, lumbar vertebra	2.65
PDB-10	French-Canadian	No	M	79	1.55	1	Right humerus	1.17
PDB-11	French-Canadian	Yes	M	75	1.68	2	Right pelvis, left femur	7.70
PDB-12	French-Canadian	No	M	66	0.98	3	Right femur, both radius	4.70
PDB-13	French-Canadian	No	F	64	1.39	1	Left femur	6.00
PDB-14	French-Canadian	Yes	F	66	0.84	1	Both occipital and parietal regions	3.67
PDB-15	French-Canadian	Yes	F	67	1.73	5	Left pelvis, sacrum, right femur, left fibula, right humerus	17.00
PDB-16	French-Canadian	Yes	M	58	3.19	6	Left pelvis, sacrum, L1 vertebra, left scapula, right tibia, skull	19.22
PDB-17	French-Canadian	Yes	F	78	1.79	1	Skull	11.00
PDB-18	French	Yes	M	58	1.22	2	Right pelvis, D7 vertebra	4.8

The normal range was 30–120 U/L

*sALP* serum phosphatase alkaline, ×* ULN* number of times of the upper limit of normal range

^a^For total sALP levels, values are × ULN ± SD

### Phenotype–genotype associations

PDB patients carrier of a *SQSTM1/P392L* post-zygotic mutation had a statistically significant lower number of affected bones than patients carrying a germinal mutation (2.2 ± 1.4 vs. 5.2 ± 4.3 affected bones, respectively, *p* = 0.002). These patient carriers of a post-zygotic mutation also had a lower Renier’s index than patients carrying a germinal mutation (8.2 ± 5.7 vs. 15.7 ± 13.4, *p* = 0.043), suggesting a lower disease extent (Table 3). Within the group of PDB patient carriers of a post-zygotic mutation, those with a familial form of the disease had 3.0 ± 2.35 affected bones, compared to 1.92 ± 0.86 for unrelated PDB patients, suggesting that familial cases had a more extensive disease. However, these results are only descriptive considering the small sample size. Although not statistically significant, PDB patients with post-zygotic mutations tended to have a lower age at diagnosis than patients without any mutations, and a higher age than patients carrying a germinal mutation. Moreover, patient carriers of a post-zygotic mutation tended to have a higher total sALP level than non-mutated patients, but lower than patients with a germinal mutation. These results suggested that patients with a *SQSTM1/P392L* post-zygotic mutation have an intermediate clinical phenotype, between patient carriers of a *SQSTM1/P392L* germinal mutation and patients with PDB non-carrier of any *SQSTM1* mutation.

**Table 3:** Comparisons of main clinical parameters between PDB patients with *SQSTM1/P392L* post-zygotic mutations and patients with germinal mutations, or patients without any *SQSTM1* mutation

	Categories of patients			Comparison of post-zygotic mutation carriers to germinal mutation carriers	Comparison of post-zygotic mutation carriers to non-mutated patients
	Post-zygotic mutation carriers (*n* = 18)	Germinal mutation carriers (*n* = 116)	Non-mutated patients (*n* = 356)	*p* value	*p* value
Age at diagnosis (years), mean ± SD	61.2 ± 12.2	58.4 ± 11.1	62.3 ± 11.1	0.324	0.716
Total sALP levels^a^	3.2 ± 3.4	4.5 ± 8.2	2.9 ± 3.1	0.629	0.977
Number of affected bones, mean ± SD	2.2 ± 1.4	5.2 ± 4.3	2.5 ± 2.3	0.002	0.884
Renier’s index (%), mean ± SD	8.2 ± 5.7	15.7 ± 13.4	10.2 ± 8.7	0.043	0.606

*sALP* serum phosphatase alkaline, ×* ULN* number of times of the upper limit of normal range

^a^For total sALP levels, values are × ULN ± SD

### Somatic mosaicism of the *SQSTM1/P392L* post-zygotic mutation in PDB patients

**Fig. 5:**
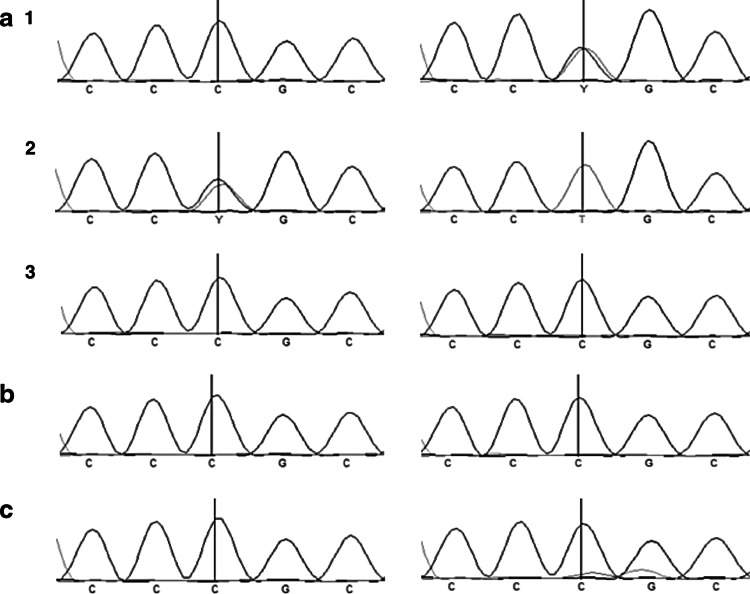
Sequencing analysis with and without the use of LNA PCR-clamping method in DNA from monocytes, lymphocytes and saliva in patients with Paget’s disease of bone (PDB). Forward short nucleotide sequence analysis of the region covering the *SQSTM1/P392L* mutation is presented. *Left panel* shows the sequences in absence of the LNA, and *right panel* shows the DNA sequences in presence of the LNA. Only one example is shown, but similar results have been observed for the three PDB patients carrier of a post-zygotic mutation. **a** DNA sequences of the monocytes population. (1) For the PDB patient carrier of a *SQSTM1/P392L* post-zygotic mutation, only the wild-type C allele was amplified in absence of the LNA, while in presence of the LNA, both wild-type C and mutant T allele were amplified. (2) For the PDB patient carrier of a *SQSTM1/P392L* germinal mutation, both wild-type C and mutant T alleles were amplified in absence of the LNA, while only the mutant T allele was amplified after the addition of the LNA in the PCR mix. (3) For the non-mutated PDB patient, only the wild-type C allele was amplified, regardless of the presence of the LNA. **b** DNA sequences of the lymphocytes population from a PDB patient carrier of a *SQSTM1/P392L* post-zygotic mutation. Only the wild-type allele C was amplified, regardless of the presence of the LNA. **c** DNA sequence of the saliva cells population from a PDB patient carrier of a *SQSTM1/P392L* post-zygotic mutation. Only the wild-type *C* allele was amplified, regardless of the presence of the LNA

Direct sequencing of PCR products from PDB patient carriers of a *SQSTM1/P392L* post-zygotic mutation showed that this mutation was present in the monocytes population, but absent in lymphocytes and saliva (Fig. 5). Similar results have been observed for the three patients, carrier of a post-zygotic mutation. The LNA PCR-clamping method performed on DNA from monocytes, lymphocytes and saliva suggested that the post-zygotic mutation was restricted to the monocytic lineage.

Among the 18 PDB patient carriers of the *SQSTM1/P392L* post-zygotic mutation, only one patient with a pagetic involvement of the pelvis was eligible for a transiliac bone biopsy. This patient was treated with an infusion of zoledronic acid 5 mg in 2008. He had normal levels of sALP and creatinine at the time of the biopsy. The X-ray of his pelvis demonstrated a typical pagetic aspect of the right pelvis with prominence of bone sclerosis, cortico-trabecular dedifferentiation and bone hypertrophy (Supplementary Fig. 1). Unfortunately, the one effort to secure active pagetic bone for laser micro-dissection proved ineffective, as the biopsy yielded tissue with insignificant cellular activity. Indeed, the osteoclastic activity appeared largely decreased in number, and slight fibrosis process has been observed in some Howship’s lacunae. An absence of osteoblast cells was also observed. Increased cortical thicknesses, architectural disorganization with heavily trabeculated bone were observed, suggesting a final ‘burn out’ phase of pagetic bone, although we cannot definitely rule out that this absence of bone cells may result from a technical problem during the fixation of the bone sample.

### Estimation of the copy number of the *SQSTM1/P392L* post-zygotic mutation

For PDB-affected patient carriers of a *SQSTM1/P392L* post-zygotic mutation, the percentage of the mutant T allele versus the wild-type C allele was ≤10 % for each DNA sample tested. For the PDB patients carrier of a heterozygous *SQSTM1/P392L* germinal mutation, this percentage was >97 %, suggesting that the mutant T allele and the wild-type C allele were present in a similar copy number, as expected. For non-mutated individuals and patients homozygous for the germinal *SQSTM1/P392L* mutation, we found about 3 % of contamination.

## Discussion

In this study, we developed an LNA PCR-clamping method reliable to detect a *GNAS/R201L* post-zygotic mutation in peripheral blood of one patient with McCune Albright syndrome, but none of FD patients, which represents a mutation rate similar to the one reported in the literature (Lietman et al. 2005). We then optimized this PCR technique to detect *SQSTM1/P92L* post-zygotic mutations in peripheral blood of patients with PDB. We found that 4.8 % of PDB patients and 1.4 % of healthy controls were carrier of this post-zygotic mutation. Among the PDB patient carriers of a post-zygotic mutation, five of them had an autosomal dominant form of the disease, but none of the relatives were carrier of a germinal or post-zygotic *SQSTM1* mutation. Since the disease-causing gene is still unknown in these families, we cannot rule out that these post-zygotic mutations can act as a modifier. Genotype–phenotype associations showed that PDB patient carriers of a *SQSTM1/P392L* post-zygotic mutation had a lower number of affected bones and a lower Renier’s index than patients carrying a germinal mutation, suggesting a less important disease extension. We did not find any statistically significant difference in the sALP levels between the different mutations groups, which is not surprising since most of patients have already been treated for PDB. Thereafter, we observed that the somatic mosaicism of *SQSTM1/P392L* mutation was restricted to the monocytic lineage. Indeed, the post-zygotic mutation was present in DNA from peripheral blood monocytes, but absent from DNA of lymphocytes and saliva. Our results, in addition to the presence of *SQSTM1/P392L* somatic mutations reported in pagetic bones (Merchant et al. 2009), confirm that *SQSTM1/P392L* post-zygotic mutations may occur in patients with PDB. The detection of *SQSTM1/P392L* post-zygotic mutations in four healthy individuals, although unexpected, may suggest that this post-zygotic mutation has an incomplete penetrance. Indeed, incomplete penetrance has also been reported for the *SQSTM1/P392L* germinal mutation, this penetrance being about 80 % by the seventh decade (Ralston and Albagha 2011). Healthy individual carriers of the *SQSTM1/P392L* germinal mutation, even after the age of 55 years and without any clinical symptoms of PDB, have also been reported in the literature (Bolland et al. 2007).

Although we were unable to confirm the presence of the *SQSTM1/P392L* post-zygotic mutation in an affected bone, we observed that the presence of this mutation was restricted to monocytes, the osteoclasts precursors. Since osteoclasts are the primary cells affected by PDB, we may hypothesize that a subset of cells originating from the monocytic lineage and carrying this mutation could contribute focally to the development of PDB. However, we cannot rule out the possibility that osteoclasts or their precursors may have recirculated from an affected bone in the peripheral blood. It will be interesting to determine if these monocytes are expressing MVNP to investigate the relation between post-zygotic mutations and viral infection, as it was done for the *SQSTM1* germinal mutation (Kurihara et al. 2011). In the future, the generation of animal model carriers of this *SQSTM1/P392L* post-zygotic mutation would be of paramount importance to better assess the functional role of post-zygotic mutations in PDB pathogenesis.

Our study has some limitations. First, we used an LNA blocking the mutation site in the forward sense only. We also decided to focus our study only on the *SQSTM1/P392L* mutation since it is the most frequent mutation associated with PDB and the only one present in the French-Canadian population, and it is located on a mutational hotspot. More than 20 mutations in the *SQSTM1* gene have been associated with PDB, and it is likely that they can occur as well in a post-zygotic form. Then, the LNA PCR-clamping method should be adapted to other *SQSTM1* mutations reported in PDB to have a better idea of the prevalence of post-zygotic mutations in PDB. Alternatively, to facilitate the detection of post-zygotic mutations, other sensitive and more high-throughput genotyping methods, such as single-nucleotide polymorphisms arrays and/or next-generation sequencing, should be considered (Omoyinmi et al. 2014). On the other hand, this study has several strengths. First, the recruitment of this large cohort of patients with the systematic collection of clinical data is a valuable tool to study the genetic factors of PDB. Second, we developed and validated a successful PCR-clamping method, which ensures its accuracy and reproducibility in the detection of low-frequency mutations.

In the literature, post-zygotic mutations are defined as mutations that arise in development during organogenesis, at the zygote stage. These mutations are classically unilateral, as evidenced by the topographically limited and tissue or organ-restricted clinical manifestations. They usually cause the development of sporadic disease in individuals with unaffected parents (Biesecker and Spinner 2013). In this study, our data do not allow us to determine at which time the mutation arose during organogenesis, but its presence in a blood lineage may suggest an early occurrence in the development of the zygote. It is important to note that there are different classes of mosaic disorders. FD is a disorder that manifests only as a mosaicism, since there is no familial form of the disease. In fact, this disease is caused by mutations that are lethal in the embryonic development, which means that they cannot be transmitted through the germ line. Cells bearing the mutation can only survive when they are in close proximity to wild-type cells (Happle 1986). Some disorders, usually transmitted in an autosomal dominant pattern of inheritance, have also been reported in mosaic forms. Patients with such mosaicism can have affected children since the post-zygotic mutation, if present in the germ line, would be transmitted to the offspring. For example, in neurofibromatosis type 1, even if most of the affected individuals are carriers of a germinal mutation in the *NF1* gene and develop a complete disease phenotype, some individuals were reported to have clinical manifestations limited to a tissue or an organ due to the presence of post-zygotic mutations in the *NF1* gene (Biesecker and Spinner 2013; Kaplan et al. 2010). Somatic mosaicism due to post-zygotic mutations can result in a milder, borderline clinical phenotype which may vary with the proportion, usually low, of cells carrying the mutation (Forsberg et al. 2013). Thus, our results suggest that, like in neurofibromatosis type 1, pagetic patient carriers of a *SQSTM1/P392L* post-zygotic mutation would develop a less extensive bone involvement than patients harboring the germ line mutation. Indeed, it is possible that the PDB extent could be somewhat correlated to the proportion of cells carrying the *SQSTM1/P392L* post-zygotic mutation. However, we did not find any particular trend in the laterality of affected bones in patients carrying the post-zygotic mutation, since both sides were affected in most of them. In this study, all patients carrying the *SQSTM1/P392L* post-zygotic mutation had a percentage of mutant T allele ≤10 %. These results could explain why we were not able to detect this post-zygotic mutation without the addition of the LNA in the PCR mix, and suggest that our technique increases the sensibility to detect *SQSTM1/P392L* mutations in our populations. In fact, the diagnostic performance of the PNA to detect *GNAS* post-zygotic mutations in fibrous dysplasia is about 1 %, and about 0.03 % for the next-generation sequencing technique (Narumi et al. 2013). Our results are in accordance with a possible mutational spectrum at germinal, post-zygotic and somatic levels for the *SQSTM1/P392L* mutation. This mutational spectrum, including post-zygotic mutations, could explain the focal nature with the asymmetric bone distribution observed in PDB, and contribute to the variable expressivity observed in familial forms of PDB with autosomal dominant pattern of inheritance.

In conclusion, we developed a reliable LNA PCR-clamping method to detect *SQSTM1/P392L* post-zygotic mutations in peripheral blood of patients with PDB. Further studies in other populations are warranted to determine more accurately the frequency of the *SQSTM1/P392L* mutations at a germinal, post-zygotic and somatic level, respectively, and to determine how post-zygotic mutations, within the *SQSTM1/P392L* mutational spectrum, may contribute to the focal nature of pagetic bone involvement.

## Electronic supplementary material

Below is the link to the electronic supplementary material.

Supplementary material 1 (TIFF 1664 kb). **Supplementary Fig. 1** X-ray of the pelvis of a patient carrier of the *SQSTM1/P392L* post-zygotic mutation. This figure shows a typical pagetic aspect of the right pelvis with prominence of bone sclerosis, cortico-trabecular dedifferentiation and bone hypertrophy
