# Supplementary figures and images for: Detection of SQSTM1/P392L post-zygotic mutations in Paget’s disease of bone
Source: Hum Genet. 2014 Sep 21;134(1):53–65. doi: 10.1007/s00439-014-1488-3 (PMC4282700; doi:10.1007/s00439-014-1488-3)

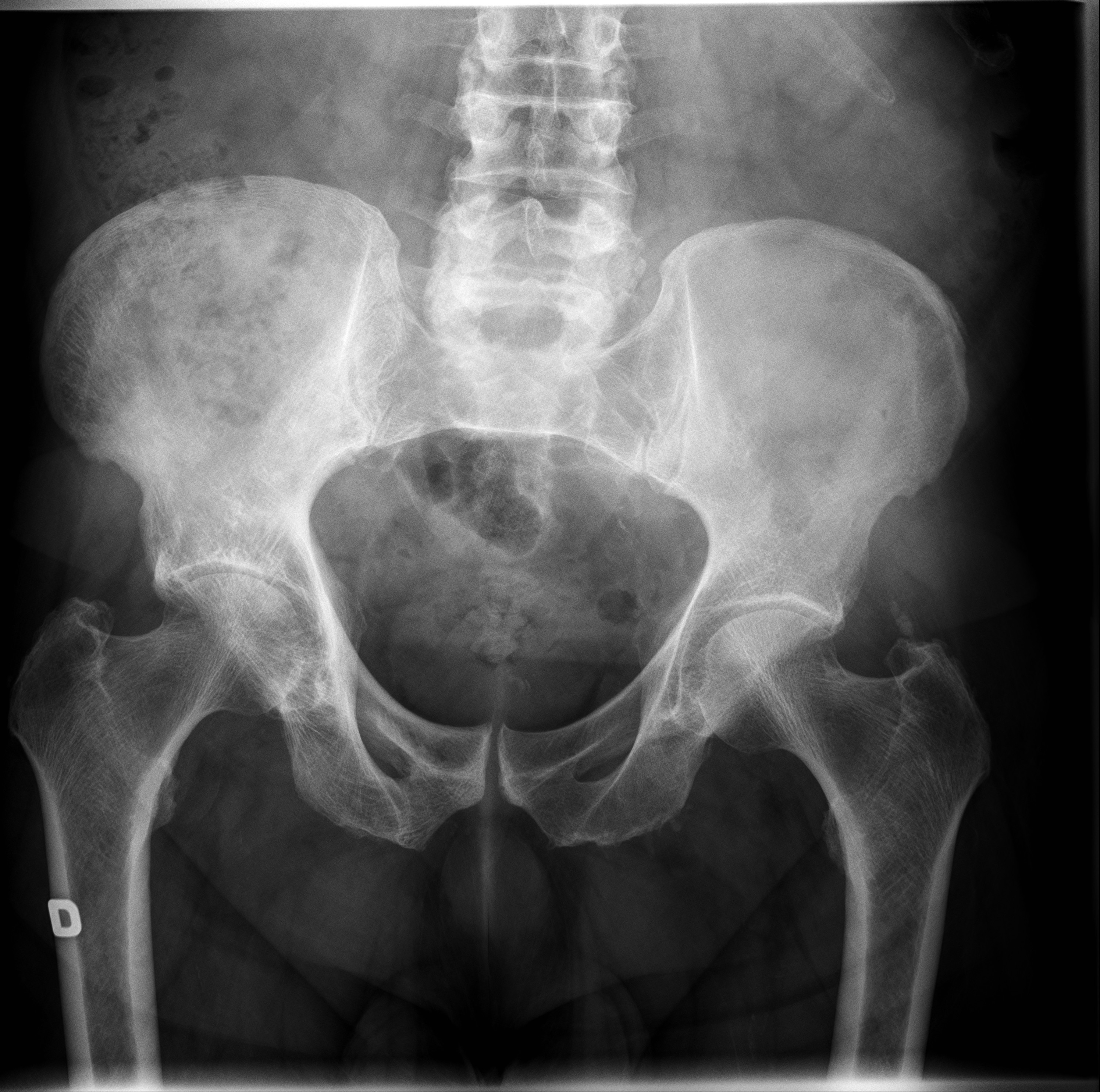

Supplement: Supplementary file 1 — Supplementary material 1 (TIFF 1664 kb). Supplementary Fig. 1 X-ray of the pelvis of a patient carrier of the SQSTM1/P392L post-zygotic mutation. This figure shows a typical pagetic aspect of the right pelvis with prominence of bone sclerosis, cortico-trabecular dedifferentiation and bone hypertrophy [file 439_2014_1488_MOESM1_ESM.tiff]
